# Vascular Reactivity to Hypercapnia Is Impaired in the Cerebral and Retinal Vasculature in the Acute Phase After Experimental Subarachnoid Hemorrhage

**DOI:** 10.3389/fneur.2021.757050

**Published:** 2022-01-13

**Authors:** Laura Warner, Annika Bach-Hagemann, Walid Albanna, Hans Clusmann, Gerrit A. Schubert, Ute Lindauer, Catharina Conzen-Dilger

**Affiliations:** ^1^Translational Neurosurgery and Neurobiology, Department of Neurosurgery, Medical Faculty, RWTH Aachen University, Aachen, Germany; ^2^Department of Neurosurgery, Medical Faculty, RWTH Aachen University, Aachen, Germany; ^3^Department of Neurosurgery, Kantonsspital Aarau, Aarau, Switzerland

**Keywords:** aneurysmal subarachnoid hemorrhage, acute phase, autoregulation, microvascular function, hypercapnia, retinal vessel analysis

## Abstract

**Objective:** Impaired cerebral blood flow (CBF) regulation, such as reduced reactivity to hypercapnia, contributes to the pathophysiology after aneurysmal subarachnoid hemorrhage (SAH), but temporal dynamics in the acute phase are unknown. Featuring comparable molecular regulation mechanisms, the retinal vessels participate in chronic and subacute stroke- and SAH-associated vessel alterations in patients and can be studied non-invasively. This study is aimed to characterize the temporal course of the cerebral and retinal vascular reactivity to hypercapnia in the acute phase after experimental SAH and compare the potential degree of impairment.

**Methods:** Subarachnoid hemorrhage was induced by injecting 0.5 ml of heparinized autologous blood into the cisterna magna of male Wistar rats using two anesthesia protocols [isoflurane/fentanyl *n* = 25 (Sham + SAH): Iso—Group, ketamine/xylazine *n* = 32 (Sham + SAH): K/X—Group]. CBF (laser speckle contrast analysis) and physiological parameters were measured continuously for 6 h. At six predefined time points, hypercapnia was induced by hypoventilation controlled *via* blood gas analysis, and retinal vessel diameter (RVD) was determined non-invasively.

**Results:** Cerebral reactivity and retinal reactivity in Sham groups were stable with only a slight attenuation after 2 h in RVD of the K/X—Group. In the SAH Iso—Group, cerebral and retinal CO_2_ reactivity compared to baseline was immediately impaired starting at 30 min after SAH (CBF *p* = 0.0090, RVD *p* = 0.0135) and lasting up to 4 h (*p* = 0.0136, resp. *p* = 0.0263). Similarly, in the K/X—Group, cerebral CO_2_ reactivity was disturbed early after SAH (30 min, *p* = 0.003) albeit showing a recovery to baseline after 2 h while retinal CO_2_ reactivity was impaired over the whole observation period (360 min, *p* = 0.0001) in the K/X—Group. After normalization to baseline, both vascular beds showed a parallel behavior regarding the temporal course and extent of impairment.

**Conclusion:** This study provides a detailed temporal analysis of impaired cerebral vascular CO_2_ reactivity starting immediately after SAH and lasting up to 6 h. Importantly, the retinal vessels participate in these acute changes underscoring the promising role of the retina as a potential non-invasive screening tool after SAH. Further studies will be required to determine the correlation with functional outcomes.

## Introduction

Aneurysmal subarachnoid hemorrhage (SAH) frequently leads to poor neurological outcomes with a high case fatality rate (1). Pathophysiological processes are complex, start early, and evolve with time, frequently causing neurological deterioration (delayed cerebral ischemia, DCI) and/or cerebral infarction with a typical delay time of 2 week after bleeding. The latest research identifies—among other factors, such as early brain injury, inflammation, microthrombosis, cortical spreading depolarization, and vasospasm—microcirculatory dysfunction with disturbance of cerebral blood flow (CBF) regulation as one of the driving mechanisms (2–5). The maintenance of a stable CBF is crucial for physiological brain function. To date, there are three key mechanisms specified: cerebral autoregulation (blood pressure-dependent cerebral vessel reaction), neurovascular coupling (local CBF regulation due to neuronal activity), and cerebrovascular reactivity to carbon dioxide (CO_2_) partial pressure [maintaining central pH and respiratory drive (6, 7)]. CO_2_ elevation (hypercapnia) in particular leads to strong vasodilatation in healthy brain tissue causing a “wash out” effect (6). For all three CBF regulation mechanisms, growing experimental (8–12) and clinical evidence of profound changes exist early and late after SAH, which are associated with the development of DCI and poor neurological outcome (2, 13). However, experimental data for the acute phase after SAH, where the complex pathophysiologic cascades accelerate, are scarce. Balbi et al. demonstrated a complete loss of CO_2_ reactivity 3 and 24 h after SAH in mice (8, 9) and similarly, Friedrich et al. demonstrated that cerebral arterioles were non-reactive to CO_2_ elevation at the same time points in rats after SAH (11). However, a detailed temporal profile of the CO_2_ reactivity as a key regulator early after SAH is still missing.

Assessment of CBF regulation after SAH in patients is still a diagnostic challenge that requires invasive and time-consuming diagnostic tools and algorithms (14) precluding this promising approach for most clinicians. As an embryologically original part of the central nervous system, the retina shares important features with the brain in morphology, vascular function, and pathophysiology (15). Furthermore, the retina and the brain both feature the three blood flow regulation mechanisms autoregulation, neurovascular coupling, and reactivity to CO_2_ elevation (16). In contrast to the brain, a considerable advantage is the comparatively easy diagnostic assessment of retinal vasculature and reactivity; new techniques, such as retinal vessel analysis, provide non-invasive and bedside direct insights on the retinal microcirculation and vasculature. Several studies report a link between retinal pathologies and neurodegenerative or cerebrovascular diseases raising expectations for a “window to the brain” (17). It has been shown that the retina participates in chronic cerebrovascular changes observed in patients with different types of dementia [for review (15)] and patients with suspected cerebral small vessel disease causing acute lacunar strokes (18). Subacute retinal changes up to 1 week following ischemic stroke (19) have also been reported. Furthermore, we recently presented the first clinical evidence of retinal vasculature changes after aneurysmal SAH. Days after SAH, retinal arteries were constricted, and neurovascular coupling was impaired compared to healthy controls. These changes were reversible in parts 3 mo after SAH indicating a time-dependent process of the observed alterations (20–22).

However, it is unclear whether these changes occur directly after SAH in the acute phase, which is substantially important to the further course of the disease. Also, it remains to be determined whether there is a correlation between the extent of pathophysiologic changes of cerebral vasculature and retinal vasculature, an indispensable requirement for the usability of retinal vascular assessment as a surrogate for cerebral circulation. As a diagnostic assessment in the acute prehospital phase is not feasible in patients and further a simultaneous assessment of retinal and cerebrovascular changes would acquire an immense and invasive effort, these questions were transferred from bed to bench side in a translational approach.

Therefore, the aim of this study was first to characterize the time course of vascular reactivity to hypercapnia in the important acute phase after experimental SAH. Second, cerebral vasculature and retinal vasculature were simultaneously assessed to investigate the comparability of the retinal alterations in the acute alterations. Finally, the extension of disturbance of the cerebral and retinal reactivity was compared.

## Methods

### Animals

All experiments were performed in compliance with the German Animal Welfare Act and the EU Directive 2010/63. The study was approved by the national state authorities (LANUV, Recklinghausen, Germany, file reference: 84-02.04.2015.A412).

Fifty-seven male Wistar rats weighing between 314 ± 19 g (Janvier Labs, Le Genest-Saint-Isle, France) were used. The animals were kept in the animal facility of the Institute of Laboratory Animal Science of the University Hospital Aachen (quality management certified according to ISO9001:2015) in 2000P type cages with water and food available ad libitum (V1534-300, Ssniff, Soest, Germany). The light-dark cycle was set at 12 h (07:00–19:00). Room temperature was kept constant at 22 ± 2°C and humidity at 55 ± 5%. Health monitoring of animals was carried out according to the Federation of European Laboratory Animal Science Associations (FELASA) recommendations (23).

To allow the animals to sufficiently acclimate to the new environment, they underwent a 7-day adaptation period. The care and health control during the adaptation phase was performed by the animal caretakers of the Institute of Laboratory Animal Science of the University Hospital of RWTH Aachen.

### Animal Preparation and Monitoring

Surgical preparation and monitoring were conducted by the same surgeon and performed as previously described (10, 24).

Surgery started at around 8 a.m. Briefly, anesthesia was induced by isoflurane, and the animal was placed in a supine position on a heating plate to maintain a constant body temperature (BT) of 37 ± 0.5°C. Ropivacaine (Ropivacaine hydrochloride, 2 mg/ml, Fresenius Kabi, Bad Homburg, Germany) as local anesthesia was used before each skin incision. After tracheotomy for artificial ventilation, femoral artery and vein were cannulated on one side for continuously measuring arterial blood pressure (ABP) and applying intravenous (i.v.) medication, respectively. Heart rate and oxygen saturation were monitored by pulse oximetry, and blood gas analyses were performed regularly. For CBF measurement, a closed cranial window was created by thinning out the bone with a drill under constant cooling over the right parietal cortex (12 × 5 mm, the center of the window ~8 mm caudal and 2.5 mm lateral of bregma). Afterward, anesthesia was changed to ketamine/xylazine (ketamine, K: 20 mg/kg/h, xylazine, X: 2 mg/kg/h) *via* continuous i.v. infusion for the K/X—Group. For the isoflurane/fentanyl group (Iso—Group), anesthesia with isoflurane and fentanyl (0.02 mg/kg/h) was continued. Additionally, both groups received a muscle relaxant to prevent involuntary eye movements (K/X—Group: Vecuronium i.v. 7.8 mg/kg/h, Iso—Group: Pancuronium bromide i.p. 1.5 mg/kg every 2 h). Two small craniotomies were performed for EEG measurement and cisterna magna blood injection. Continuous intracranial pressure (ICP) measurement was implemented *via* a needle inserted in the cisterna magna through the atlanto-occipital membrane.

For SAH induction, 0.5 ml of heparinized autologous blood, withdrawn from the femoral artery catheter, was injected over 1 min into cisterna magna as previously described (10). Sham animals underwent the same procedure as SAH animals except for the blood injection. ABP, EEG, and ICP were measured and recorded continuously during the whole observation period (starting 30 min pre-SAH for baseline record and continued until 6 h after SAH). At the end of the observation period after 6 h, the animals were sacrificed in deep anesthesia by i.v. injection of potassium chloride (2.5 M). Brains were removed, inspected, and documented for successful SAH induction.

### Experimental Design

#### Anesthetic Protocols and Group Design

As isoflurane is a potent vasodilator by itself and has been reported to increase the retinal blood flow by 29% (25), the experiments were performed following two established anesthetic protocols: isoflurane/fentanyl (Iso—Group) and ketamine/xylazine (K/X—Group) summing up in 4 groups: Iso—Group Sham, Iso—Group SAH, K/X—Group Sham, and K/X—Group SAH (**Figure 2**). With this approach, we were able to analyze cerebral and retinal blood flow reactions to hypercapnia starting from two different base levels in health and disease. Sample size calculation was performed by *a priori* power analysis (G^*^Power 3.1.7) with alpha = 0.05 and power (1-beta) = 0.80, based on previous experience with comparable study design. Twenty-five animals were included in the Iso—Group (Sham *n* = 9; SAH *n* = 16). In the K/X—Group, 32 animals were included (Sham *n* = 16, SAH *n* = 16; **Figure 2**). Experiments of the Iso—Group were performed first, followed by K/X—Group with randomization between Sham and SAH in this group only.

#### CBF—Recording

**Figure 1:**
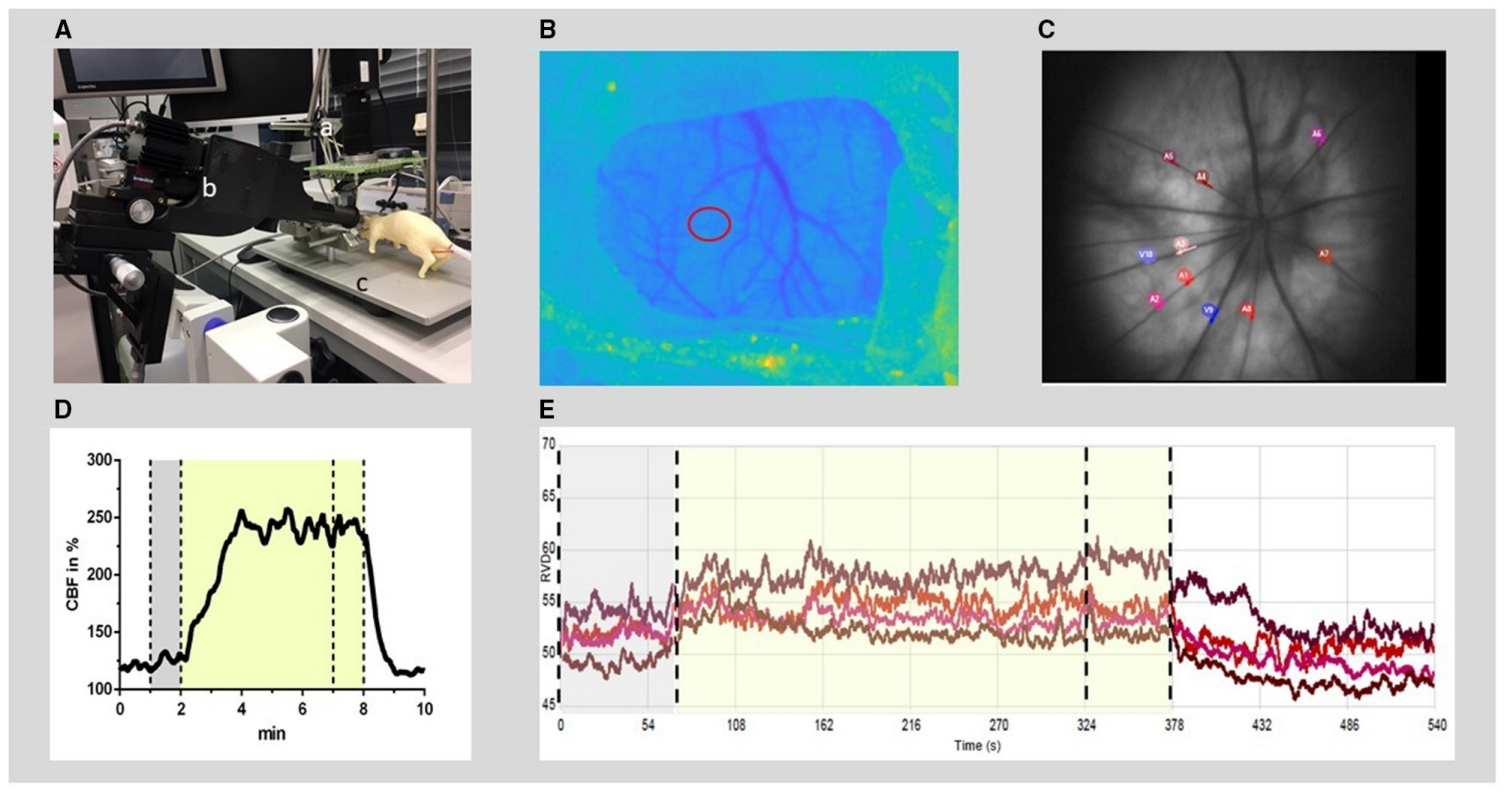
Experimental setup for RVD and CBF recording and offline analysis. **(A)** Experimental setup: the rat (dummy rat used for illustration) was positioned in a stereotactic frame (c) under a superficial tissue imaging system (a) and CBF was calculated from continuously recorded raw images of laser speckle contrast. A retinal vessel analyzer (b) was positioned in front of the left eye. RVDs were recorded intermittently during hypercapnia challenges induced by hypoventilation. **(B)** Anatomical image at the cranial window (thinned bone) with the region of interest for CBF time course calculation (red circle). **(C)** Example of a fundus image: 4–8 arteries were selected (red and purple circles) for offline analysis of RVD from each video sequence. **(D)** Typical time course of CBF during the hypercapnic period at baseline. **(E)** Example of diameter analysis of retinal vessels at baseline, performed by the commercial RCrodent Imedos- software. **(D,E)** The time period of hypercapnia is marked by the transparent yellow area, dotted lines indicate the recording period used for baseline (gray) and hypercapnic response calculation, respectively. RVD, retinal vessel diameter; CBF, cerebral blood flow.

Cerebral blood flow was measured, recorded, and evaluated as previously described (10). Briefly, animals were placed in a prone position under a prototype superficial tissue imaging system (STIS, Biomedical Optics Laboratory, RheinAhrCampus, Remagen, Germany). Data processing was performed offline. CBF was calculated from raw images of laser speckle contrast within a region of interest positioned over the somatosensory cortex at an area of microcirculation devoid of larger pial vessels (Figure 1). For CBF analysis, an initial baseline was recorded for 5 min with the further course of CBF normalized to this baseline. The increase during the triggered hypercapnia was calculated as the percentage change from the respective actual baseline immediately before the hypercapnic phase.

#### Retinal Vessel Diameter (RVD)—Recording

To measure the RVD at selected time points, the camera system RCrodent from Imedos (IMEDOS Systems UG, Jena, Germany) was used for dynamic vessel analysis. To dilate the pupil, a mydriatic (Pharma Stulln GmbH, Stulln, Germany) was applied to the left eye, and a lens (Ocular Instruments, Bellevue, WA, USA) was positioned in front of the eye using a micromanipulator. The eye was regularly moistened with a transparent protective gel to avoid dehydration of the cornea. Illumination (530 nm) was set to a maximum of 30 lux. Videos of the retinae were recorded over a 9-min period (1 min baseline, 5 min hypercapnia, and 3 min regression phase). Vessel dilations were calculated offline. From each video sequence, four to eight arterial vessels were selected with a mean distance from the optic nerve head (ONH) of 2.5 × ONH plexus diameters, and diameter analysis (expressed in arbitrary units) was automatically performed by the commercial RCrodent Imedos-software (Figure 1). In each animal, the artery with the best reactivity at baseline was selected for subsequent analysis at later time points in Sham or after SAH. Due to variations in the quality of the fundus recordings between the subsequent hypercapnia periods, it was not always possible to record from identical vessel segments within each experiment over time. Reactivity to hypercapnia was expressed as percentage change from the individual baseline preceding each period of hypercapnia.

#### Hypercapnia

To evaluate the reactivity of cerebral and retinal vasculature to CO_2_ elevation, hypercapnia challenges were performed at several predefined time points: at baseline before SAH, and 30, 60, 120, 240, and 360 min after SAH, respectively, after the start of the 360 min recording period in Sham animals. Cerebral vasoreactivity and retinal vasoreactivity to hypercapnia were measured simultaneously in each animal. Measurements of RVD were exclusively performed at these time points, while measurement of CBF, EEG, ICP, and ABP was continuously recorded. Before each hypercapnia challenge, an arterial blood gas analysis was performed to document pCO_2_ and pO_2_. Each hypercapnia period lasted 9 min, after 1 min of measuring under physiological conditions, hypoventilation was started by reducing the respiratory rate by 20 breaths/min to induce hypercapnia. To prevent hypoxia, the O_2_ supply was simultaneously increased. After 4 min, blood gas analysis was performed again to verify the CO_2_ elevation. One minute later, the respiratory rate was increased back to normoventilation, followed by another 3 min of recording of the recovery phase of the vessels.

### Statistical Analysis

For statistical analysis and graph design, GraphPad Prism (versions 9.1.1 and 9.1.2) was used. Data were tested for normal distribution *via* the Shapiro-Wilk test or Kolmogorov-Smirnov test. Statistical comparisons within each group against baseline were performed by 2-Way-ANOVA or mixed-model-ANOVA, if data points were missing, with time as the dependent factor and treatment (Iso—Group Sand SAH, K/X—Group Sham and SAH) as the independent factor, followed by Dunnett's multiple comparisons test and by Sidak's test for comparison of CBF with RVD reactivity, respectively. For comparison of vascular reactivity to CO_2_ elevation between retinal and cerebral vasculature in each group, reactivity was normalized to baseline for each group and vascular bed, respectively. In rare cases of comparably small reactivity at baseline, the normalization procedure may result in a disproportional and thus erroneously high percentage change even when only small changes of the original data occurred. Therefore, after normalization, Grubbs test with *p* < 0.01 was performed to identify outliers: Nine outliers out of 234 data points were removed as suggested by the test (0 of 36 in Iso-CBF, 3 of 36 in Iso-RVD, 2 of 78 in K/X-CBF, and 4 of 84 in K/X-RVD). The same test to identify outliers performed on the original (not-normalized) data set did not suggest excluding any data. Data written in the text are presented as median [first quartile to the third quartile]. In the figures, data are presented as boxplots with median and 25 and 75% percentile as boxes and data range as whiskers (**Figures 3**, **4**) or as median as symbol and 25 and 75% percentile as whiskers (**Figure 5**). A *p* < 0.05 was considered as significantly different.

## Results

### Animals and ICP and CBF Courses in SAH Groups

**Figure 2:**
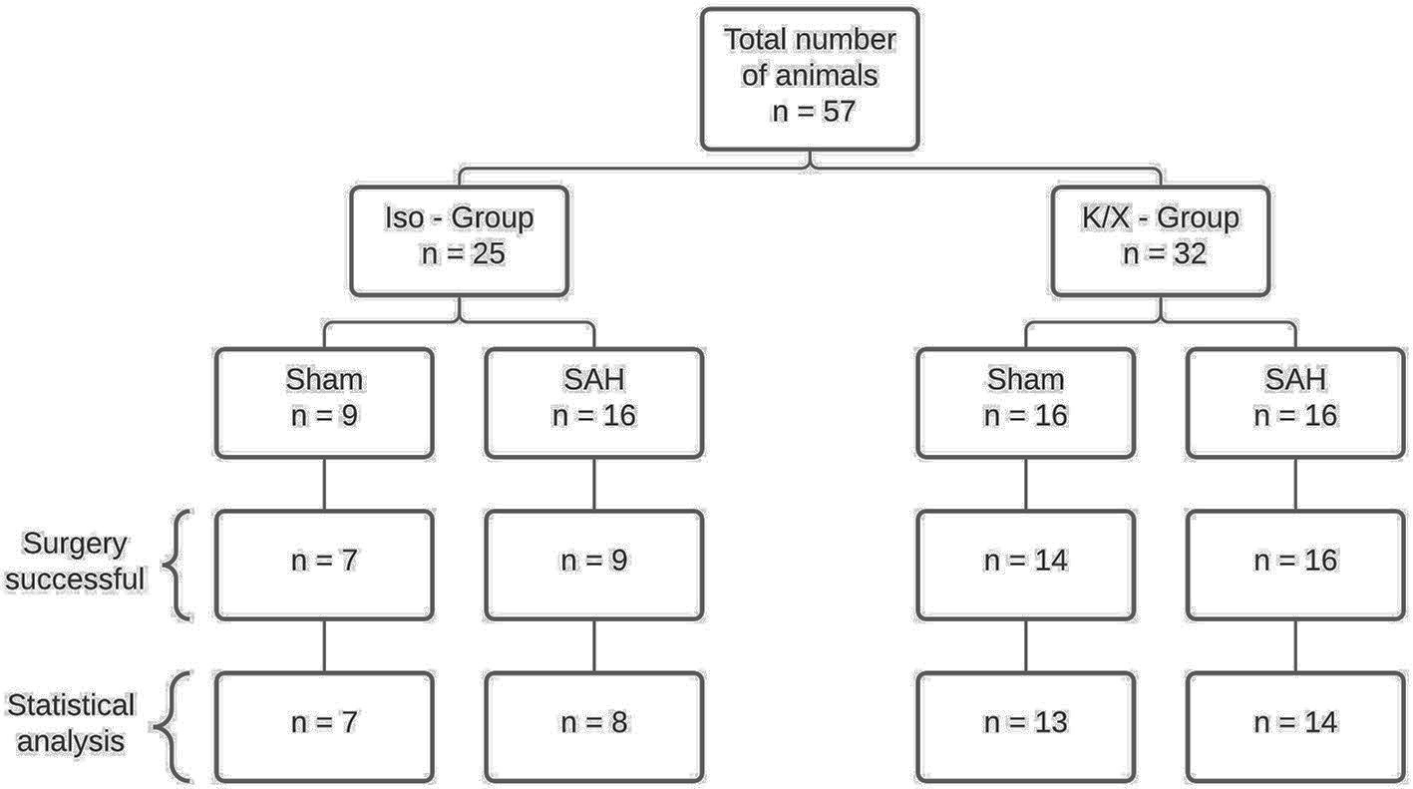
Diagram of sample size distribution. A total of *n* = 57 animals were allocated to two different anesthesia groups. Within the group, a distinction was made between Sham and SAH. A total of 42 animals were included for statistical analysis. Reasons for exclusion were death during surgery (*n* = 11), failed SAH induction (*n* = 3), and uncontrollable bleeding from the surgery wound at the cranial window with distortion of CBF measurement (*n* = 1); SAH, subarachnoid hemorrhage; Iso, isoflurane; K/X, ketamine/xylazine.

A total of 57 rats were randomly assigned to two different anesthetic groups. For data analysis, the following criteria had to be fulfilled to ensure successful SAH induction: increase in ICP and/or drop in CBF, and assessment of brains after SAH. A total of *n* = 15 animals were excluded for death during surgery (*n* = 11), failed SAH induction (*n* = 3), and uncontrollable bleeding from the surgery wound at the cranial window with distortion of CBF measurement (*n* = 1; Figure 2). The ICP showed the typical course known from other studies of the group (10, 24), presenting a high peak at the end of the 1 min blood-injection period, followed by a moderate, yet still significantly elevated plateau phase throughout the observation period (except for ICP in Iso—Group at 360 min), with no significant difference between both anesthesia protocols (Supplementary Figure 1A). With CBF starting from the baseline of 100%, after a transient severe drop to ischemic values at the time of the ICP peak (Iso—Group SAH: 6.1% [5.5–14.6], *p* < 0.0001; K/X—Group SAH: 9.3% [7.2–21.8], *p* < 0.0001; no significant difference between groups), CBF reached normal values again within 15 min in the K/X—Group and within 60 min in the Iso—Group, respectively. Further-on, a slight albeit significant hyperemia was detectable at 240 min (129.2% [116.6–137.6], *p* = 0.0198) and 360 min [136.3% [124.0–146.9], *p* = 0.0310) in K/X—Group, whereas a mild decrease in CBF occurred at the end of the measurement under Iso-anesthesia (70.8 [62.2–83.4], *p* = 0.0101). Significant differences occurred between both anesthesia protocols at 30 min (*p* = 0.0464) and 360 min (*p* = 0.0016; Supplementary Figure 1B).

### Physiological Parameters

Arterial blood gas analyses (BGA; pO_2_, pCO_2_, pH), mean ABP, heart rate (HR), O_2_ saturation (SpO_2_), and BT remained within physiological ranges throughout the measurement (except for blood gases during hypercapnia; Table 1). While comparing the data at baseline with the data at the end of the measurement for each parameter, a slight but significant reduction in ABP occurred in the Iso-SAH-group (*p* = 0.047) and the K/X-Sham-group (*p* = 0.0242). The reduced values are still well above the lower limit of pressure autoregulation, therefore an impact on vascular reactivity to hypercapnia can be ruled out.

**Table 1:** Physiological parameters at baseline and after 6 h.

	**Iso—Group, baseline**		**K/X—Group, baseline**	
	**Sham**	**SAH**	**Sham**	**SAH**
pO_2_ (mmHg):	151 [130–166]	140 [125–141]	107 [103–130]	129 [109–157]
pCO_2_ (mmHg)	38.6 [28.2–40.4]	35.4 [32.3–42.8]	40.5 [36.8–46.3]	39.1 [33.8–46.3]
pH:	7.38 [7.37–7.40]	7.39 [7.37–7.40]	7.37 [7.33–7.39]	7.38 [7.36–7.42]
ABP (mmHg):	74 [63–83]	90 [70–104]^*^	89 [78–97]^+^	83 [76–93]
BT (°C):	37.3 [37.0–37.5]	37.4 [36.9–37.4]^#^	37.8 [37.3–37.9]^#^	37.4 [37.0–37.6]
HR (bpm):	337 [311–359]	379 [346–420]	344 [305–405]	354 [305–429]
SpO_2_ (%):	99 [99–99]	99 [99–99]	99 [99–99]	99 [99–99]
	**Iso—Group, 6 h**		**K/X—Group, 6 h**	
	**Sham**	**SAH**	**Sham**	**SAH**
pO_2_ (mmHg):	145 [116–161]	145 [106–186]	132 [114–142]	124 [114–146]
pCO_2_ (mmHg)	35.4 [31.7–39.4]	37.2 [34.0–38.3]	41.9 [39.4–44.5]	41.3 [37.1–45.6]
pH:	7.36 [7.33–7.38]	7.38 [7.37–7.39]	7.36 [7.34–7.37]	7.36 [7.35–7.39]
ABP (mmHg):	74 [69–78]	76 [70–81]^*^	82 [70–85]^+^	77 [73–83]
BT (°C):	37.5 [37.3–37.5]	37.2 [37.0–37.6]	37.3 [37.1–37.7]	37.5 [37.2–37.7]
HR (bpm):	347 [331–363]	349 [306–369]	367 [332–419]	319 [295–347]
SpO_2_ (%):	99 [99–99]	99 [99–99]	99 [99–99]	99 [99–99]

*All parameters remained within physiological ranges during the measuring period. ABP, mean arterial blood pressure; BT, body temperature; HR, heart rate in beats per minute; SpO_2_, systemic oxygen saturation*;

*,+ *p < 0.05 for bl vs. 6 h in the respective group*;

# *p < 0.05 between respective groups at baseline*.

### Verification of Hypercapnia

**Figure 3:**
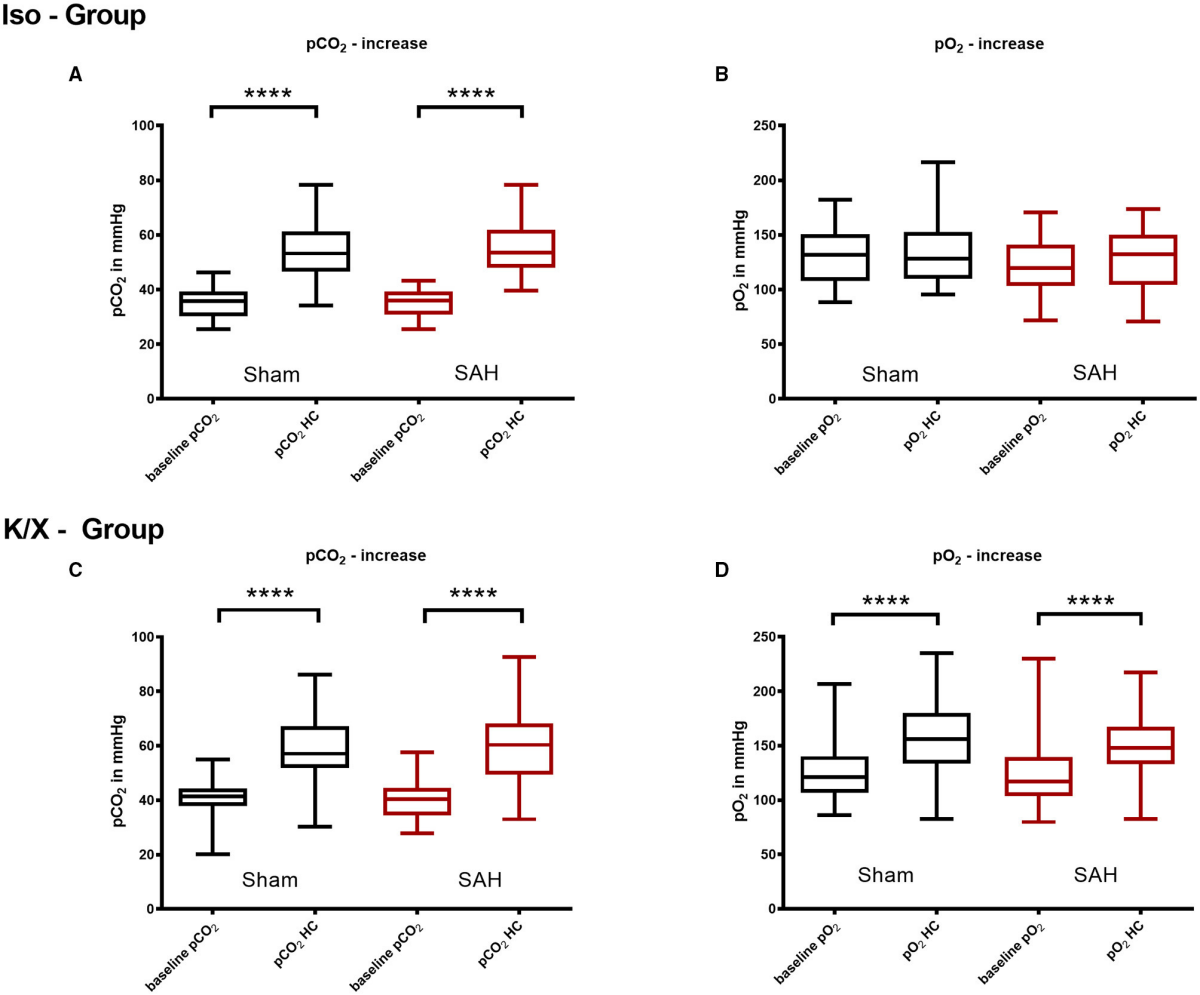
Arterial pCO_2_ and pO_2_ analysis in hypercapnia periods. Blood gas analysis was performed before and during hypercapnia. In the Iso—Group there was a significant pCO_2_ increase during hypercapnia **(A)**, while pO_2_ did not change significantly **(B)**; during hypercapnia, both pCO_2_ **(C)** and pO_2_ **(D)** increased significantly in the K/X—Group; SAH, subarachnoid hemorrhage; Iso, isoflurane; K/X, ketamine/xylazine; HC, hypercapnia; boxes show median and 25 and 75% percentile and whiskers show data range; ^****^p < 0.0001.

During hypoventilation, there was a significant increase of pCO_2_ compared to baseline values in both SAH and Sham groups (K/X—Group: Sham 41.3 mmHg [20.2–44.4] vs. 57.1 mmHg [30.3–57.1], *p* < 0.0001, SAH 40.3 mmHg [34.5–44.6] vs. 60.3 mmHg [49.4–68.2], *p* < 0.0001, Iso—Group: Sham 39.7 mmHg [36.2–43.8] vs. 56.4 mmHg [50.8–65.6], *p* < 0.0001, SAH 36.0 mmHg [30.9–39.3] vs. 53.5 mmHg [48.0–61.9], *p* < 0.0001), with no difference in CO_2_ elevation between groups. In the Iso—Group, the pO_2_ remained stable (Sham 131.7 mmHg [108.0–150.8] vs. 128.3 mmHg [109.7–152.6], SAH 119.8 mmHg [103.4–140.9] vs. 132.2 mmHg [104.2–150.0]), whereas it was increased significantly in the K/X—Group (Sham 120.9 mmHg [106.8–140.39] vs. 155.8 mmHg [133.4–180.1], *p* < 0.0001, SAH 117.2 mmHg [103.6–139.3] vs. 147.8 mmHg [133.1–167.2], *p* < 0.0001; Figure 3).

### Cerebral Vascular Reactivity to Hypercapnia

In both anesthetic protocols, cerebral CO_2_ reactivity in Sham-operated animals was stable over the observation period (Supplementary Figure 2).

**Figure 4:**
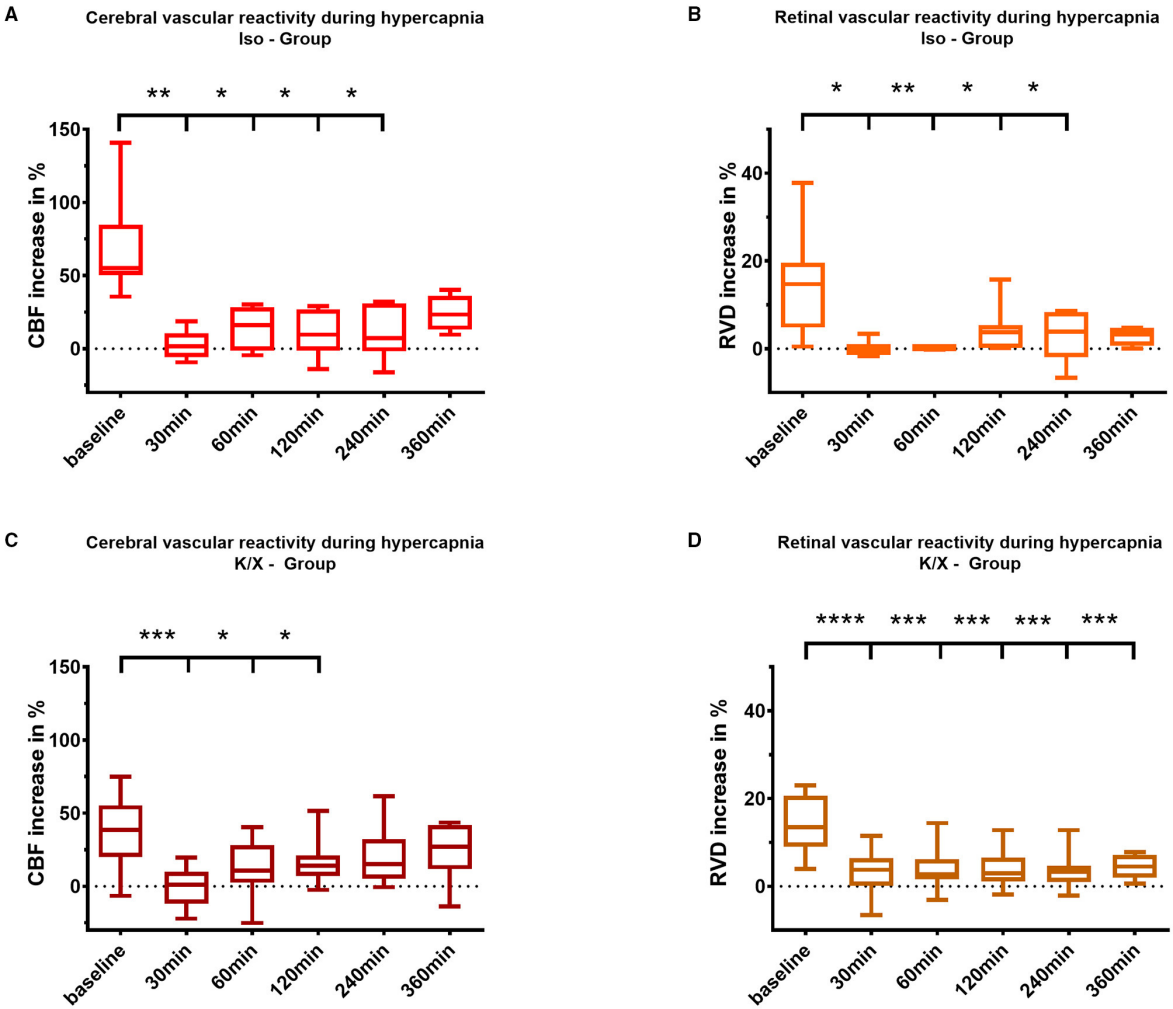
Cerebral and retinal vascular reactivity to hypercapnia after SAH. Iso—Group: Cerebral vascular reactivity **(A)** as well as retinal vascular reactivity **(B)** to hypercapnia were impaired up to 240 min after SAH; K/X—Group: Cerebral CO_2_ reactivity was disturbed up to 120 min after SAH **(C)**, whereas impairment of retinal vessels persisted throughout the whole measurement period of 360 min **(D)**. SAH, subarachnoid hemorrhage; Iso, Isoflurane; K/X, Ketamine/Xylazine; CBF, cerebral blood flow; RVD, retinal vessel diameter; boxes show median and 25% and 75% percentile and whiskers show data range; ^*^p < 0.05, ^**^p < 0.01, ^***^p < 0.001, ^****^p < 0.0001.

In the Iso—Group SAH, cerebral CO_2_ reactivity was immediately impaired compared to baseline starting at 30 min after blood injection (baseline 55.2% [50.2–84.7] vs. 30 min 1.8% [−5.8–10.5], *p* = 0.0090) and lasting up to 240 min (240 min 7.3 % [−1.8–30.9], *p* = 0.0136). In the K/X—Group SAH, impairment of cerebral CO_2_ reactivity was comparable, however, with a faster recovery compared to Iso anesthesia. CO_2_ response was significantly impaired up to 120 min compared to baseline (baseline 38.5% [19.9–55.4] vs. 30 min 1.1% [−11.8–10.1], *p* = 0.0003; 120 min 14.0% [7.1–21.0], *p* = 0.0469; Figure 4).

### Retinal Vascular Reactivity to Hypercapnia and Analysis of Resting Diameters

Retinal CO_2_ reactivity in Sham-operated animals was stable over the observation period with a slight albeit significant increase of CO_2_ reaction in the K/X—Group after 120 min.

Following SAH, similar to the cerebral vasculature, an impaired vascular reactivity to CO_2_ of the retinal vasculature was observed in both anesthesia groups. Under isoflurane, retinal vasculature showed disturbed CO_2_ reactivity during hypercapnia, even with a tendency toward vasoconstriction immediately after SAH (baseline 15.5% [9.6–19.9] vs. 30 min −0.4% [−1.4–1.1], *p* = 0.0135), and a reduced response lasting up to 240 min compared to baseline (240 min 3.9% [−1.9–8.6], *p* = 0.0263). In the K/X—Group SAH, retinal CO_2_ reactivity was significantly impaired over the whole observation period compared to baseline (baseline 13.43% [9.0–20.7] vs. 30 min 3.8% [0.1–6.4], *p* < 0.0001, vs. 360 min 4.5% [2.0–7.2], *p* = 0.0001; Figure 4).

For the cerebral circulation, it is known from this and our previous studies (10, 24) that with isoflurane anesthesia strong hypoperfusion occurs up to 2 h after SAH induction in this model. We, therefore, compared the resting diameters of the retinal vessels (in arbitrary units), taken directly before the hypercapnic challenges, at baseline with each time point thereafter in Sham or SAH. The diameters remained stable over the observation period in Sham and, in contrast to the known changes in CBF, we did not detect a significant change after SAH either (Supplementary Figure 3).

### Comparison of Vascular Reactivity to CO_2_ Elevation Between Retinal and Cerebral Vasculature

**Figure 5:**
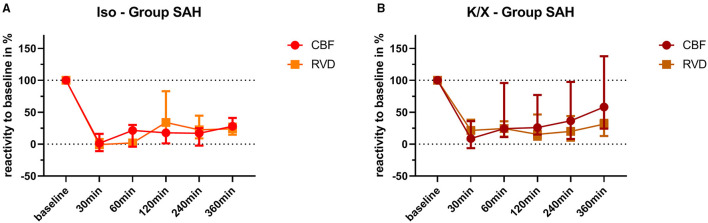
Comparison of vascular reactivity to hypercapnia between retinal and cerebral vasculature. Reactivity at baseline was normalized to 100% for each vascular bed in each animal, and the changes in percentage were calculated over time after SAH. Averaged data of both vascular beds of each group are depicted for comparison. No significant differences were found in the Iso—Group **(A)** and in the K/X—Group **(B)** for all time points of hypercapnia challenge. SAH, subarachnoid hemorrhage; Iso, isoflurane; K/X, ketamine/xylazine; CBF, cerebral blood flow; RVD, retinal vessel diameter; symbols show median and whiskers show 25 and 75% percentile.

Retinal and cerebral reactivity to hypercapnia was compared to assess whether they are comparably affected (Figure 5). For this purpose, reactivity was normalized to baseline for each group and vascular bed, respectively. In the Iso—Group, reactivity to hypercapnia in both cerebral and retinal vasculature was comparably and strongly disturbed up to 360 min after SAH induction, with a transient recovery of the retinal but not of the cerebral vasculature at 120 min. Under K/X anesthesia, the reactivity to hypercapnia in both the cerebral and retinal vasculature was again at least transiently disturbed up to 120 min after SAH induction. Retinal hypercapnia response remained persistently impaired, whereas reactivity in the cerebral circulation transiently recovered at 60 min (*p* = 0.1894), was impaired again at 120 min (*p* = 0.0064), and permanently recovered thereafter until the end of the measurement phase. While comparing retinal with cerebral reactivity at each time point in each group, no statistically significant difference was observed.

## Discussion

Our experimental study on vascular reactivity to hypercapnia in the acute phase of SAH documents an immediate and pronounced disturbance of this key component of blood flow regulation both in the cerebral and the retinal vasculature. After SAH, the disturbance was observed as early as 30 min after induction and lasted for several hours in both compartments indicating the participation of the retinal vasculature in acute pathophysiologic alterations primarily affecting the brain. Notably, after normalization to baseline, not only a simultaneous and parallel behavior of both vascular beds but also a comparable extent of impairment could be observed. Additionally, we found that the two different anesthetic protocols neither influence the extent of impaired hypercapnia reactivity nor the temporal pattern except for a faster recovery of CBF in the K/X group.

Previous studies on the hypercapnia reactivity in the acute phase after experimental SAH also reported a significant impairment, albeit with a larger extent of disturbance: Friedrich et al. found that after SAH, the pial and cortical tissue microcirculation were non-reactive to CO_2_ after 3 and 24 h in mice and rats (11). Similarly, in additional studies from the same group, Balbi et al. also described a complete loss of CO_2_ reactivity after 3 and 24 h after SAH in mice (8, 9). In our study, we detected a significant reduction of the cerebrovascular reactivity, with a complete loss observed in some animals especially at the earliest time points. Depending on the anesthesia protocol, the CO_2_ reactivity in our study in rats was significantly reduced up to 2 or 4 h, respectively, with a recovery toward the end of the observation time of 6 h. Of note, there are inherent differences between the studies regarding the anesthesia protocol [combination of medetomidine/midazolam/fentanyl in mice (8, 9, 11), chloral hydrate in rats (11) vs. isoflurane/fentanyl or ketamine/xylazine in our study], the rat strain [Sprague Dawley (11) vs. Wistar in our study], and the SAH model [filament perforation model (8, 9, 11) vs. blood injection model in our study]. Thus, albeit a direct comparison to our data is impeded, the occurrence of significantly disturbed hypercapnia reactivity under variable experimental conditions points toward a universal pathophysiological alteration of the cerebral vasculature early after SAH.

Until now, experimental data about impaired hypercapnia reactivity during the hyperacute phase (within the first hour) and at more prolonged time points (up to 6 h) after SAH and data about retinal reactivity to CO_2_ after SAH, in general, are lacking. Our study is, therefore, the first to report a distinct temporal pattern of vascular CO_2_ reactivity in the acute phase after SAH both within the cerebral and the retinal vascular bed, with immediate impairment lasting over several hours.

There are several issues from our findings worthy of discussion: vascular dysfunction starts immediately after bleeding. This short time period possibly indicates the importance of the ictus itself with a massive ICP peak within the first minutes after bleeding, followed by a moderate plateau, with no significant difference between both anesthesia protocols. However, it seems unlikely that the elevated ICP during the plateau phase contributes to the reduced vascular responses to hypercapnia. After a transient severe drop to ischemic values at the time of the ICP peak, the CBF reached normal values again within 15 min in the K/X-Group and within 60 min in the Iso-Group, respectively. In addition, it has been shown that CBF and blood volume responses to functional activation remained preserved during moderately elevated ICP (26). We, therefore, assume that a primarily pressure induced impact on the blood supply to the brain and on cerebrovascular reactivity can be ruled out. A metabolic response evoked by blood degradation products in the subarachnoid space also seems more improbable in view of the immediate reaction. Based on our knowledge of the dependence of the cerebral vasculature on a basal nitric oxide (NO) availability for physiological neurovascular coupling and CO_2_ reactivity (27–29), functional impairment of vascular reactivity by scavenging of perivascular NO by—at this early time point still intact—erythrocytes seems reasonable (30). Wang et al. observed a decrease of CO_2_ reactivity after regional SAH caused by the punctuation of a single pial arteriole after 3 h (31). This makes an additional impact on the subarachnoid blood itself possible. Besides the blood-cell-induced reduction of perivascular NO, cortical spreading depolarization waves have to be considered as a further possible mechanism, as the hypercapnic response has been shown to be severely reduced for hours after a single CSD wave in healthy animals (32). The frequent occurrence of CSDs has recently been shown in the acute phase after SAH in mice (33) and has to be further evaluated in our SAH model in rats.

An ultra-early and at least sub-acutely protracted inability of the cerebral vasculature to properly react to hypercapnia might be of interest in translational research. A conceivable therapeutic potential of the vascular reactivity to CO_2_ after SAH will only be of success when the cerebral vasculature is physiologically reacting to changes of arterial pCO_2_. Results from experimental studies in mice show that CO_2_ reactivity recovers in the chronic phase 1 mo after SAH (34). In a clinical phase 1 study, CO_2_ as a potent vasodilator was used aiming at increasing CBF therapeutically on days 4–14 after SAH (35). A recently published follow-up study investigating the optimum duration of the hypercapnic challenge for CBF elevation again suggested hypercapnia as a promising approach for perfusion enhancement in the critical phase of DCI (36). Whether this potentially improves outcome remains to be determined. Importantly, spontaneous hyperventilation and consecutive cerebral hypoperfusion are associated with DCI and poorer neurological outcomes (37), underlining the crucial role of profound knowledge on the ability or disability of the cerebral vasculature to react to CO_2_ after SAH.

Therefore, an easily applicable bedside method for the assessment of cerebrovascular reactivity would be highly desirable. In the present experimental study, we demonstrate for the first time that the retinal vascular bed participates in the acute dysfunction of the cerebral vasculature after SAH not only in temporality but also in the degree of the observed disturbance. Only while using K/X as an anesthesia regime, there was a discrepancy between the retinal and the cerebral reactivity toward the end of the observation period, with persistent impairment of the retinal hypercapnic response throughout the measurement period while the CBF reactivity already recovered within 240 min. The reason for this difference is not known so far, however, specific mechanisms of the anesthetic drugs may be involved. Ketamine has been shown to be neuroprotective (38) and to reduce the occurrence of cerebral spreading depolarization in the acute phase after SAH (33) which may both specifically protect the neurovascular unit in the brain. Another explanation of the prolonged RVD reduction in the K/X-Group may be that isoflurane and ketamine/xylazine differently affect the intraocular pressure and associated functions, such as the scotopic threshold responses (39). However, due to methodological reasons, we did not additionally measure the intraocular pressure in our study. In summary, we think more data are needed to determine whether there is a truly prolonged impairment in the retinal vessels at time point 360 min or whether it is rather a narcotic effect.

Our findings of an overall parallel impairment of the hypercapnic reactivity of the retinal and cerebral vasculature extend the previous knowledge about the retina as a window to the brain in chronic neurodegenerative diseases, such as Alzheimer's and inaugurate, a potential new monitoring method as a non-invasive diagnostic tool for acute neurological diseases. From an embryological point of view, the retina is part of the central nervous system, more precisely of the diencephalon. The vascular supply of the retina is provided by the ophthalmic artery, thus sharing important autoregulatory mechanisms, such as CO_2_ reactivity or neurovascular coupling with strictly intracranial vessels. The optic nerve and the ophthalmic artery within its sheath directly connect the retina with the brain: Of note, in SAH, between 12 and 23% of the patients develop a vitreous/ and or subhyaloid hemorrhage (Terson's Syndrome). The pathophysiology is mostly but not exclusively linked to the raised ICP in the moment of bleeding (40). Furthermore, the latest research indicates a glymphatic system not only in the brain but in the retina (41) assuming perivascular pathways through which vasoactive agents of the subarachnoid space may also directly reach the retinal microvasculature. In summary, acute changes of retinal vascular reactivity in SAH seem reasonable. Our findings underscore the latest clinical research, where altered RVD and impairment of neurovascular coupling days after SAH were observed in patients. Importantly, changes were in parts reversible at the time of follow up implying an acute but transient effect of SAH on the retinal vasculature (20–22). However, information about the degree of parallel CBF impairment in these patients is lacking precluded by the invasive and extensive character of the necessary diagnostic procedures. Our experimental data show that in simultaneous measurement of CBF and RVD, both vessel beds behave in a parallel way in rats in the early phase after SAH. This supports the assumption that retinal vessel analysis may hold the key for a possible new, non-invasive, and bedside diagnostic tool enabling live vascular assessment in SAH. However, the clinical relevance in terms of DCI and neurological outcome of these findings still needs to be proven. In addition, further studies are needed to evaluate possible common mechanisms of impairment in both vascular beds for example by the spread of blood degradation products within the glymphatic space (41) or by autonomous nervous system dysfunction (42) in the acute and a chronic course of the disease.

### Limitations

Our complex experimental setup with extensive surgical preparation on the one hand and the delicate and easily vulnerable brain and retinal tissue requiring careful preparation and maintenance on the other hand entailed a comparatively high drop-out rate of 26%. The drop-out rate was accentuated in the Iso—Group SAH causing comparatively few data points especially at the end of the observation period. This may have led to a sample size bias with a higher probability of effect inflation (43).

For the experiments, two different anesthesia protocols were used with the Iso—Group performed first, using our well-established regime (10, 24), resulting in significant increases to hypercapnia in the retinal and the cerebral vasculature, albeit with some variability between the animals. It is well-known that isoflurane itself dilates systemic and cerebral vasculature, probably being partly responsible for the variability. In addition, the isoflurane-induced dilation has been shown to be even larger in the retinal vasculature compared with the cerebral vasculature (44). To rule out that our finding is influenced by the type of anesthesia, we repeated the study while using a common protocol of ketamine/xylazine combination, which has been shown to significantly reduce the retinal blood flow at unstimulated conditions (45). Due to this sequential procedure, we were not able to randomize between the two anesthesia protocols, which may have introduced a possibly existing, albeit probably only small, bias.

Similar to every animal model, the single injection model used in this study has some important limitations itself. Especially the missing vessel perforation mimicking the human SAH in having an injured vessel with direct hemorrhagic brain lesions is an important drawback. The double hemorrhage model mimics best the cerebral vasospasm and delayed ischemic lesions and, therefore, seems to be more suitable to study the delayed effects of SAH (46, 47). However, we choose the single hemorrhage model instead of the perforation model because of better control of the subarachnoid blood amount resulting in a more comparable acute brain injury (SAH severity) and lower mortality rates.

Hypercapnia was achieved by controlled hypoventilation for a predefined time period in all animals. Simultaneously, the O_2_ supply was slightly increased to prevent hypoxia. This procedure was verified *via* blood gas analysis for every single measure period. In both KX—Groups, there was a slight but significant increase of arterial pO_2_ during hypercapnia. However, this moderate hyperoxia is not expected to influence functional vascular reactivity at all (48, 49).

## Conclusion

This study demonstrates fundamental changes of one important CBF regulation mechanism in the acute phase after SAH in two vascular beds: the cerebral and the retinal vascular reactivity to hypercapnia is deeply impaired. Pathologic alterations start immediately after SAH and last up to several hours. Importantly, the retinal vasculature not only participates in these acute changes of altered cerebral vascular reactivity to hypercapnia but behaves in a parallel way regarding the degree of vascular impairment and the temporal course.

With this bedside to bench approach in form of a simultaneous assessment of CBF and retinal vessel reactivity after SAH, our results support and extend first data in patients with human SAH, which showed retinal vessel alterations several days after SAH. This underscores the potential role of the retina as a non-invasive, bedside screening tool even for highly acute neurological diseases. Further studies will be required to determine the long-time changes after experimental SAH and correlation with functional outcome.

## Data Availability Statement

The raw data supporting the conclusions of this article will be made available by the authors, without undue reservation.

## Ethics Statement

The animal study was reviewed and approved by Landesamt für Natur, Umwelt und Verbraucherschutz (LANUV) Nordrhein—Westfalen, Recklinghausen, Germany (file reference: 84-02.04.2015.A412) in line with the EU Directive 2010/63/EU on the protection of animals used for scientific purposes, and was performed in accordance with the ARRIVE Guidelines.

## Author Contributions

CC-D, GS, and UL: conceived and designed the experiments and the study protocol. LW, AB-H, WA, UL, and CC-D: constructed experimental set up. LW: performed the experiments. LW, CC-D, and UL: analyzed the data, interpretation of the data, and illustrations. CC-D and LW: first drafting of the manuscript. AB-H, HC, GS, WA, UL, and CC-D: critical review of the manuscript. All authorship requirements have been met and the final manuscript was critically revised and approved by all authors.

## Funding

This work was supported by the grants from the START – Program of the Faculty of Medicine RWTH Aachen University for CC, German Research Foundation (Deutsche Forschungsgemeinschaft DFG, grant number LI 588/5-1, LI 588/5-2) as part of the research unit ‘‘Severity assessment in animal based research'' FOR2591 for UL, and 50% financial support for the RCrodent from Imedos (IMEDOS Systems UG, Jena, Germany) by the Faculty of Medicine RWTH Aachen University for UL.

## Conflict of Interest

The authors declare that the research was conducted in the absence of any commercial or financial relationships that could be construed as a potential conflict of interest.

## Publisher's Note

All claims expressed in this article are solely those of the authors and do not necessarily represent those of their affiliated organizations, or those of the publisher, the editors and the reviewers. Any product that may be evaluated in this article, or claim that may be made by its manufacturer, is not guaranteed or endorsed by the publisher.
