# Supplementary material for: Vascular Reactivity to Hypercapnia Is Impaired in the Cerebral and Retinal Vasculature in the Acute Phase After Experimental Subarachnoid Hemorrhage
Source: Front Neurol. 2022 Jan 13;12:757050. doi: 10.3389/fneur.2021.757050 (PMC8793938; doi:10.3389/fneur.2021.757050)
Supplement: Supplementary file 1 [file Data_Sheet_1.docx]

Supplementary Material

Figure 1. ICP and CBF courses in SAH – Groups.
(A.1, A.2) The ICP showed a high peak at the end of the one minute blood-injection period, followed by a moderate, yet still significantly elevated plateau phase throughout the observation period (except for ICP in Iso – Group at 360min) (A.2), with no significant difference between both anesthesia protocols (A.1); (B.1, B.2) Unstimulated CBF showed a transient severe drop to ischemic values at the time of the ICP-peak and reached normal values again within 15min in the K/X – Group and within 60min in the Iso – Group, respectively. Further-on, a slight albeit significant hyperaemia was detectable at 240min and 360min in K/X – Group, whereas a mild decrease of CBF occurred at the end of the measurement under Iso-anaesthesia (B.2). Significant differences occurred between both anaesthesia protocols at 30min and 360min (B.1). ICP = intracranial pressure, CBF = cerebral blood flow; Iso = Isoflurane, K/X = Ketamine/Xylazine; boxes show median and 25% and 75% percentile and whiskers show data range; * p<0.05, ** p<0.01, *** p<0.001, **** p<0.0001, A.1, B.1: comparison between groups at each time point; A.2, B.2: comparisons to baseline.

Figure 2. Cerebral and retinal vascular reactivity to hypercapnia in Sham – Group. Hypercapnic reactivity of cerebral blood flow (A = Iso, C = K/X) as well as retinal CO_2_ reactivity (B = Iso, D = K/X) in sham operated animals was stable over the observation period with a slight albeit significant increase of CO_2_ reaction in the K/X - Group after 120 min in the retinal vasculature (D). Iso = Isoflurane, K/X = Ketamine/Xylazine, CBF = cerebral blood flow, RVD = retinal vessel diameter; boxes show median and 25% and 75% percentile and whiskers show data range; * p<0.05.

Figure 3. Resting vessel diameter in the retina.
We compared the resting diameters of retinal vessels (in arbitrary units), taken directly before the hypercapnic challenges, at baseline with each time point thereafter in sham or SAH. The diameters remained stable over the observation period in sham and after SAH. Iso = Isoflurane, K/X = Ketamine/Xylazine, RVD = retinal vessel diameter; boxes show median and 25% and 75% percentile and whiskers show data range.

Table 1. Statistical analysis of cerebral and retinal vascular reactivity to hypercapnia. Data were tested for normal distribution. Statistical comparisons within each group against baseline were performed by 2-Way-ANOVA or mixed- model- ANOVA, if data points were missing followed by Dunnett’s multiple comparisons test. Iso = Isoflurane, K/X = Ketamine/Xylazine, CBF = cerebral blood flow, RVD = retinal vessel diameter, SAH = subarachnoid hemorrhage

| **Group** | **Median** | **n** | **p-value** |
| --- | --- | --- | --- |
|  | [q1-q3] |  | (vs. baseline) |
|  |  |  |  |
| **Iso - Group CBF Sham** | | | |
| baseline | 78.61 [54.4- 114.5] | 7 | - |
| 30min | 54.98 [49.5- 65.0] | 5 | 0.4735 |
| 60min | 72.01 [53.2- 115.0] | 7 | 0.9997 |
| 120min | 65.27 [44.5- 74.5] | 7 | 0.3160 |
| 240min | 49.89 [30.8- 73.9] | 7 | 0.2817 |
| 360min | 29.81 [20.3- 36.6] | 3 | 0.2709 |
| **Iso - Group CBF SAH** | | | |
| baseline | 55.23 [50.2- 84.7] | 8 | - |
| 30min | 1.84 [-5.8- 10.5] | 7 | **0.0090** |
| 60min | 16.3 [-1.2- 28.2] | 5 | **0.0412** |
| 120min | 9.67 [-1.2- 27.0] | 6 | **0.0140** |
| 240min | 7.29 [-1.8- 30.9] | 6 | **0.0136** |
| 360min | 23.35 [13.1- 36.1] | 4 | 0.2100 |
| **Iso - Group RVD Sham** | | | |
| baseline | 11.99 [6.2- 21.0] | 7 | - |
| 30min | 9.91 [6.6- 12.2] | 5 | 0.7326 |
| 60min | 17.6 [9.7- 18.0] | 7 | 0.9998 |
| 120min | 20.00 [11.2- 22.5] | 7 | 0.9997 |
| 240min | 17.83 [11.6- 20.1] | 7 | 0.8427 |
| 360min | 16.66 [8.9- 16.7] | 3 | 0.9999 |
| **Iso - Group RVD SAH** | | | |
| baseline | 15.51 [9.6- 19.9] | 8 | **-** |
| 30min | -0.40 [-1.4- 1.1] | 7 | **0.0135** |
| 60min | 0.28 [0.02- 0.5] | 5 | **0.0043** |
| 120min | 3.86 [1.5- 8.0] | 6 | **0.0252** |
| 240min | 3.90 [-1.9- 8.4] | 6 | **0.0263** |
| 360min | 4.02 [1.7- 4.7] | 4 | 0.0998 |
| **K/X - Group CBF Sham** | | | |
| baseline | 42.85 [24.3- 66.4] | 12 | - |
| 30min | 39.92 [27.0- 69.3] | 12 | 0.9974 |
| 60min | 29.99 [22.5- 57.2] | 12 | 0.8415 |
| 120min | 26.93 [13.6- 66.7] | 12 | 0.9602 |
| 240min | 37.93 [19.5- 86.9] | 12 | 0.9486 |
| 360min | 27.79 [8.8- 33.7] | 12 | 0.3504 |
| **K/X - Group CBF SAH** | | | |
| baseline | 43.53 [26.1- 56.6] | 13 | - |
| 30min | 2.40 [-7.6- 10.1] | 13 | **0.0003** |
| 60min | 11.17 [3.1- 28.6] | 13 | **0.0453** |
| 120min | 12.40 [5.3- 18.0] | 13 | **0.0469** |
| 240min | 16.0 [5.2- 33.6] | 13 | 0.3208 |
| 360min | 26.45 [9.1- 41.9] | 13 | 0.3590 |
| **K/X - Group RVD Sham** | | | |
| baseline | 15.14 [8.7- 15.5] | 13 | - |
| 30min | 8.59 [5.2- 18.0] | 13 | 0.9405 |
| 60min | 8.13 [3.5- 15.5] | 13 | 0.0878 |
| 120min | 8.43 [4.5- 13.9] | 13 | **0.0479** |
| 240min | 7.11 [4.5- 11.7] | 13 | 0.1982 |
| 360min | 8.33 [3.0- 12.3] | 13 | >0.9999 |
| **K/X - Group RVD SAH** | | | |
| baseline | 13.43 [9.0- 20.7] | 14 | - |
| 30min | 3.77 [0.1- 6.4] | 14 | **<0.0001** |
| 60min | 2.745 [1.6- 6.2] | 14 | **0.0004** |
| 120min | 2.93 [1.1- 6.6] | 14 | **0.0008** |
| 240min | 3.36 [1.0- 4.7] | 14 | **0.0003** |
| 360min | 4.51 [2.0- 7.2] | 14 | **0.0001** |

Table 2. Statistical analysis of comparison of vascular reactivity to hypercapnia between retinal and cerebral vasculature. Data were tested for normal distribution. Statistical comparisons between were performed by 2-Way-ANOVA or mixed- model- ANOVA if data points were missing, followed by Sidak’s test. Iso = Isoflurane, K/X = Ketamine/Xylazine, CBF = cerebral blood flow, RVD = retinal vessel diameter, SAH = subarachnoid hemorrhage

| reactivity compared to baseline | | | | | | |
| --- | --- | --- | --- | --- | --- | --- |
| **Group** | **Median** | **n** | **Group** | **Median** | **n** | **p-value** |
|  | [q1-q3] |  |  | [q1-q3] |  | (CBF vs. RVD) |
|  |  |  |  |  |  |  |
| **Iso - Group CBF SAH** | | | **Iso - Group RVD SAH** | | | |
| baseline | 100 [100- 100] | 8 | baseline | 100 [100- 100] | 8 | - |
| 30min | 1.30 [-11.1- 16.1] | 7 | 30min | -0.78 [-6.3- 8.1] | 6 | >0.9999 |
| 60min | 21.46 [-4.1- 29.9] | 5 | 60min | 1.90 [-0.1- 3.6] | 5 | 0.7277 |
| 120min | 17.68 [1.1- 22.7] | 6 | 120min | 33.86 [18.4- 83.0] | 6 | 0.5350 |
| 240min | 16.93 [-2.3- 25.1] | 6 | 240min | 22.34 [-9.0- 44.5] | 5 | 0.8946 |
| 360min | 27.94 [25.9- 41.1] | 4 | 360min | 24.13 [14.8- 32.2] | 3 | 0.8816 |
| **K/X - Group CBF SAH** | | | **K/X - Group RVD SAH** | | | |
| baseline | 100 [100- 100] | 13 | baseline | 100 [100- 100] | 14 | - |
| 30min | 8.85 [-6.4- 36.0] | 13 | 30min | 21.40 [6.9- 38.3] | 13 | 0.8771 |
| 60min | 24.2 [11.0- 95.7] | 13 | 60min | 24.48 [11.8- 35.7] | 13 | 0.6604 |
| 120min | 25.9 [15.0- 77.0] | 13 | 120min | 15.26 [6.9- 46.5] | 13 | 0.7059 |
| 240min | 36.62 [7.9- 97.4] | 12 | 240min | 19.93 [5.4- 43.9] | 14 | 0.6856 |
| 360min | 58.21 [24.2- 137.6] | 13 | 360min | 31.31 [12.8- 38.1] | 13 | 0.1277 |
