# Supplementary figures and images for: Vascular Reactivity to Hypercapnia Is Impaired in the Cerebral and Retinal Vasculature in the Acute Phase After Experimental Subarachnoid Hemorrhage
Source: Front Neurol. 2022 Jan 13;12:757050. doi: 10.3389/fneur.2021.757050 (PMC8793938; doi:10.3389/fneur.2021.757050)

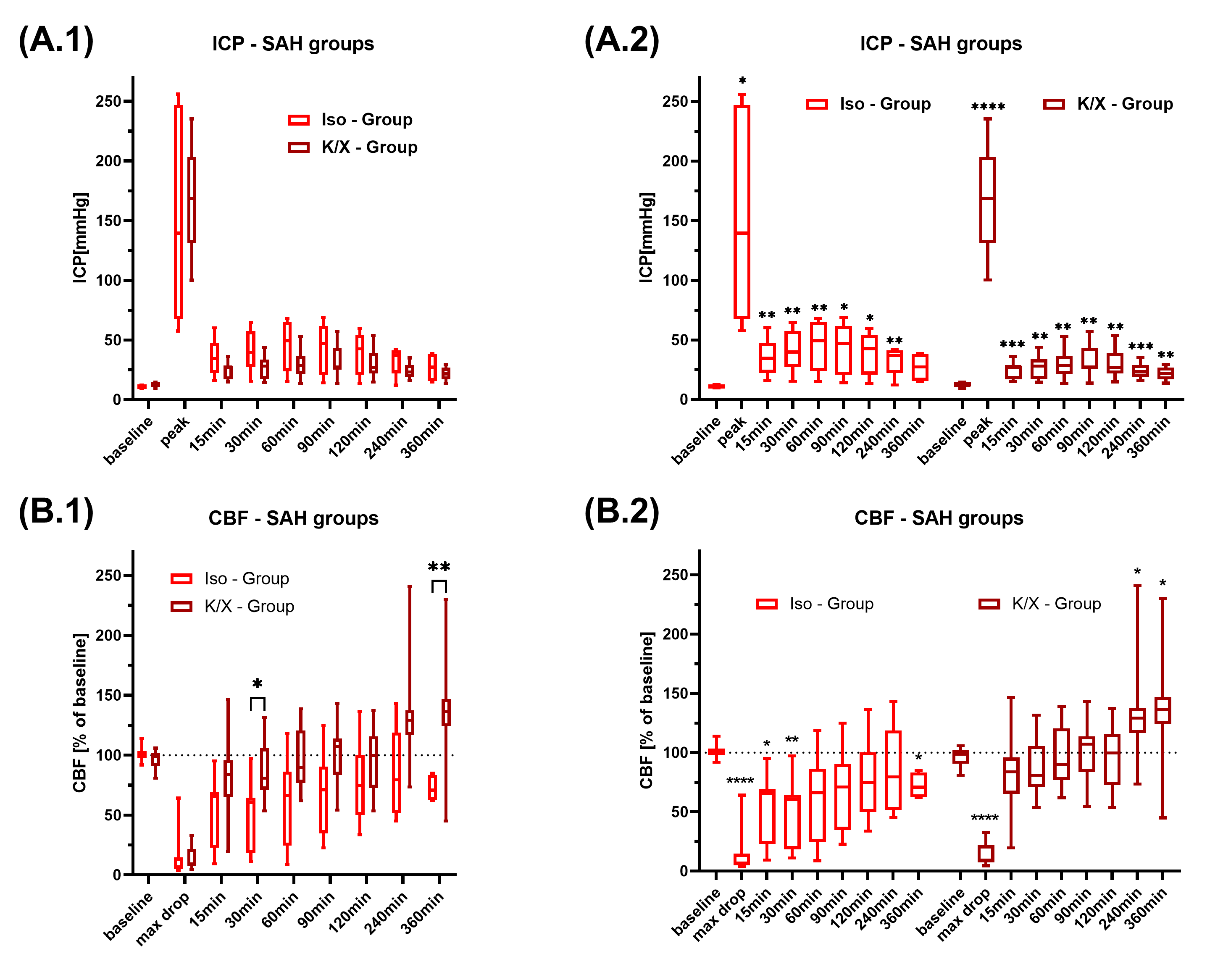

Supplement: Supplementary file 2 [file Image_1.TIF]

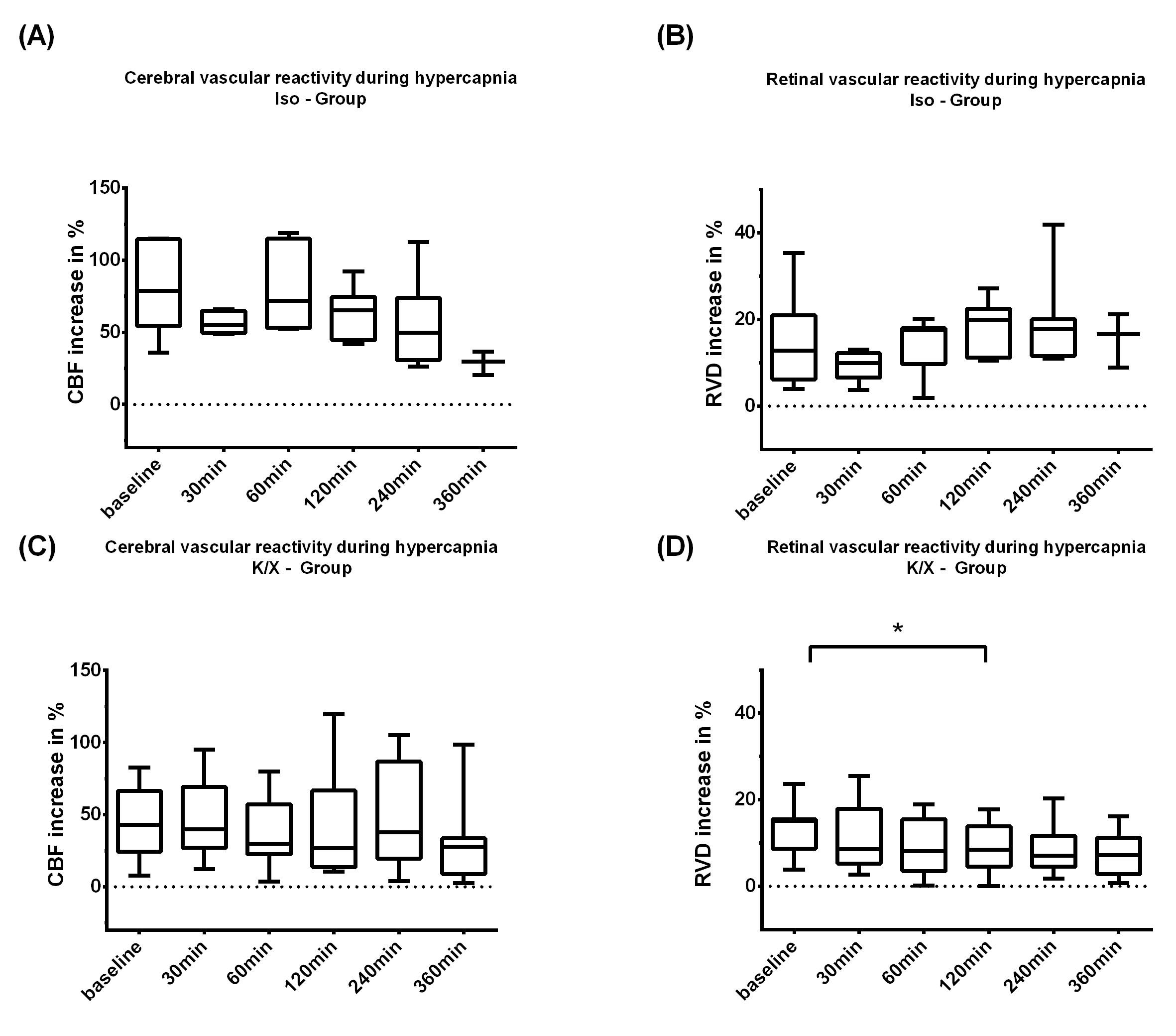

Supplement: Supplementary file 3 [file Image_2.TIF]

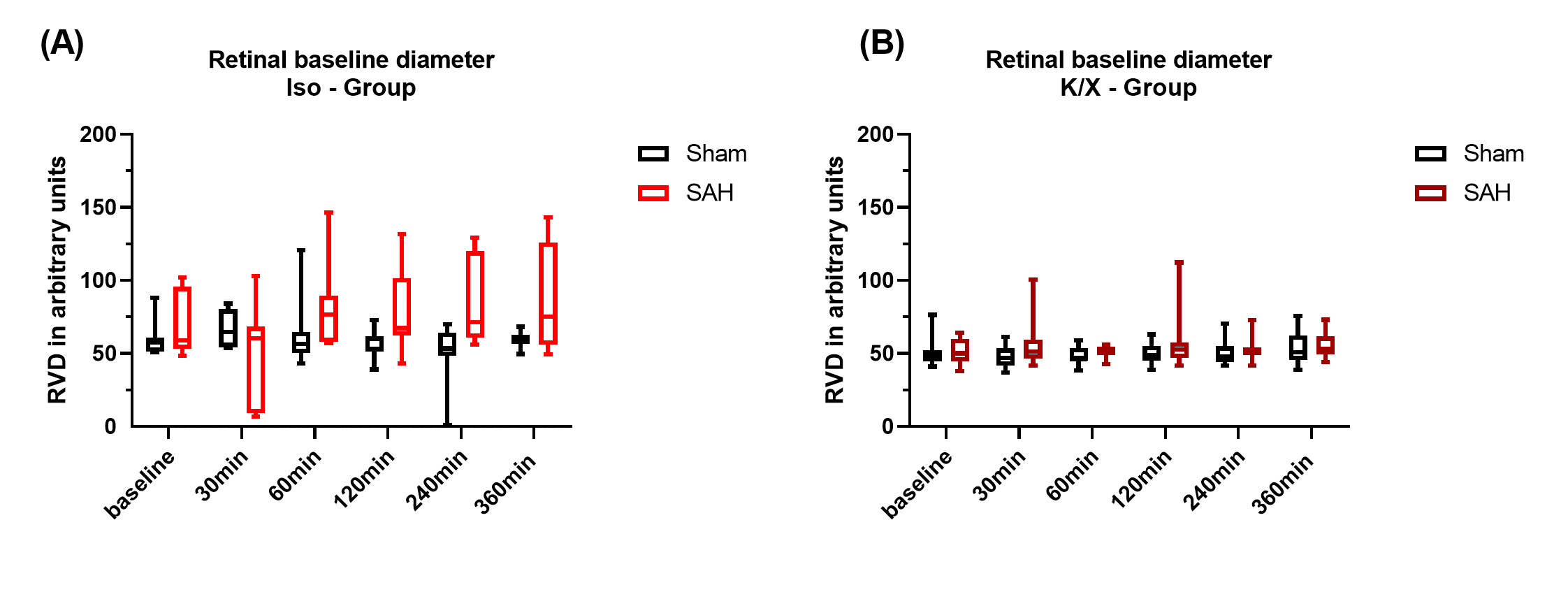

Supplement: Supplementary file 4 [file Image_3.TIF]
